# Development of Low-Cost Instrumentation for Single Point Autofluorescence Lifetime Measurements

**DOI:** 10.1007/s10895-017-2101-7

**Published:** 2017-05-25

**Authors:** João Lagarto, Jonathan D. Hares, Christopher Dunsby, Paul M. W. French

**Affiliations:** 1 Photonics Group, Department of Physics, Imperial College London, London, SW7 2AZ UK; 2 Kentech Instruments Ltd., Howbery Park, Wallingford, OX10 8BD UK

**Keywords:** Autofluorescence, Fluorescence lifetime, Low-cost, Time-resolved fluorescence spectroscopy, Fibre-optic

## Abstract

Autofluorescence lifetime measurements, which can provide label-free readouts in biological tissues, contrasting e.g. different types and states of tissue matrix components and different cellular metabolites, may have significant clinical potential for diagnosis and to provide surgical guidance. However, the cost of the instrumentation typically used currently presents a barrier to wider implementation. We describe a low-cost single point time-resolved autofluorescence instrument, exploiting modulated laser diodes for excitation and FPGA-based circuitry for detection, together with a custom constant fraction discriminator. Its temporal accuracy is compared against a “gold-standard” instrument incorporating commercial TCSPC circuitry by resolving the fluorescence decays of reference fluorophores presenting single and double exponential decay profiles. To illustrate the potential to read out intrinsic contrast in tissue, we present preliminary measurements of autofluorescence lifetime measurements of biological tissues ex vivo*.* We believe that the lower cost of this instrument could enhance the potential of autofluorescence lifetime metrology for clinical deployment and commercial development.

**Electronic supplementary material:**

The online version of this article (doi:10.1007/s10895-017-2101-7) contains supplementary material, which is available to authorized users.

## Introduction

Autofluorescence spectroscopy provides a non-invasive label-free approach to characterise biological tissues ex vivo and in vivo and has shown potential to report structural and biochemical changes associated with pathological transformations. Since fluorescence lifetime measurements of biological tissue measure the fluorescence intensity dynamics, they provide robust readouts that are effectively ratiometric in a single spectral channel, which is important in heterogeneous, optically scattering samples, and can provide additional (functional) information to that available from intensity measurements alone [1, 2]. Time-resolved fluorescence spectroscopy (TRFS) can thus overcome common problems associated with fluorescence intensity measurements including variations in excitation and collection efficiency and can discriminate between fluorophores with overlapping fluorescence emission spectra but different decay times, as typically occurs with tissue autofluorescence [3]. In addition, the fluorescence lifetime can be affected by the fluorophore’s microenvironment and therefore its measurement can provide a readout of environmental parameters such as temperature [4], oxygenation [5] or pH [6] through the use of an appropriate fluorophore, which may be a specialised exogenous probe. Further information can be obtained by fitting fluorescence decay profiles to more complex models [7–9] or using phasor analysis [10, 11]. TRFS of autofluorescence has been shown to discriminate between healthy and diseased tissues in the case of different types of cancer [12–17], atherosclerosis [18–20], myocardial infarction [21] or cartilage degradation [22]. It may also be applied to engineered tissues [23] and could provide a useful monitor for bioreactors.

A common concern associated with fluorescence lifetime measurements is the complexity, size and cost of the instrumentation, which can limit the wider application of TRFS, particularly in clinical settings, where compact and user-friendly devices are required [2]. Over the last decade, however, a number of advances have been reported that could facilitate the wider uptake of fluorescence lifetime metrology and imaging, including for clinical research. These include the development of compact illumination sources [24, 25], low-cost detection electronics for both time-domain [26, 27] and frequency-domain [28, 29] measurements and integrated on-chip devices [30]. However, only a few low-cost, compact instruments, i.e. utilising affordable and compact light sources and detection instrumentation, have been reported e.g. [30–32] and their potential has not yet been demonstrated in biomedical applications. These typically employ inexpensive LEDs for illumination with gated photodiode detectors to temporally resolve the fluorescence signal. However, despite promising initial results, these instruments provide reduced temporal accuracy and scope for complex fluorescence decay analysis comparable to the “gold standard” of time-correlated single photon counting (TCSPC). The recent development of detectors exploiting complementary metal oxide semiconductor (CMOS) single photon avalanche detector (SPAD) arrays to realise TCSPC [33, 34], present new opportunities for time-resolved analysis of biological and other fluorophores. However, TCSPC is conventionally implemented with ultrashort (fs-ps) excitation pulses that require relatively expensive laser sources and there is still a lack of compact and portable autofluorescence lifetime instrumentation that can probe the complexity of autofluorescence signals that typically emanate from biological tissue.

Here we present the development of a low-cost time-resolved fluorometer implemented in a single point measurement instrument that utilises a modulated laser diode for excitation and a standard photon-counting photomultiplier tube (PMT) in combination with a home-made constant fraction discriminator (CFD) for time-resolved detection of the autofluorescence signal. The detection methodology is based on the digital frequency domain heterodyne method previously introduced by Colyer et al. [29]. In this proof-of-concept study, we aimed to demonstrate a low cost instrument and its potential to provide contrast in autofluorescent biological samples. We provide a detailed description of its design and characterisation, including a comparison against a well-characterised TCSPC-based time-resolved spectrofluorometer, previously developed in our laboratory [35].

## Theory

This low-cost time-resolved fluorometer builds on the digital frequency domain (DFD) approach introduced by Colyer et al. [29]. Briefly, the excitation laser source is pulsed at frequency *f*_exc_, and fluorescence photons are detected using a photon-counting detector, such as a PMT or SPAD. A temporal sampling window is then implemented at a slightly higher sampling frequency, *f*_*s*_, that is detuned from *f*_exc_ by the cross-correlation frequency, *f*_cc_, such that.

1 $$ f{}_{cc} = \left|{f}_s-{f}_{exc}\right| $$

**Fig. 1:**
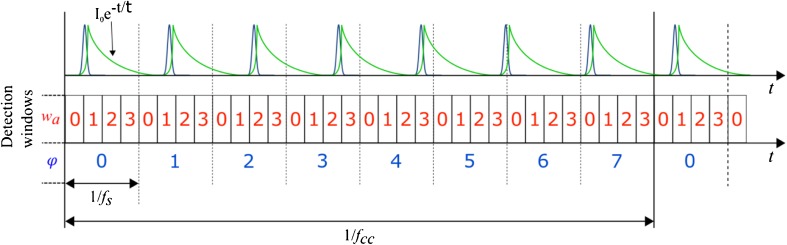
Schematic representation of the heterodyning method using 4 detection windows sliding across 7 excitation periods during a cross-correlation period 1/*f*_cc_, which corresponds to 8 sampling periods of 1/*f*_s_. When a photon is detected, it is “tagged” with a window of arrival number (in red, *w*_a_) and a phase shift counter number (in blue, *φ*), which keep track of the phase shift between excitation and sampling frequencies. The phase shift counter is reinitialised every cross-correlation period

Accordingly, the sampling window (1/*f*_s_ in Fig. 1) continuously slides through the excitation period (1/*f*_exc_) to record the fluorescence decay at different offsets relative to the excitation pulse, until both frequencies (*f*_exc_ and *f*_s_) are in phase again after time 1/*f*_*cc*_. To improve the temporal resolution, the sampling period can be divided into multiple detection windows that sample the fluorescence decay at different points. An arbitrary number of windows can be used, subject to the bandwidth of the sampling circuitry. The temporal resolution of the fluorescence acquisition improves with shorter sampling windows and the temporal sampling density can be controlled independently through the mismatch of the excitation and sampling frequencies. A schematic diagram of this technique is shown in Fig. 1.

## Hardware Implementation

This time-resolved detection approach can be fully implemented using relatively low-cost digital electronics, which can potentially increase its clinical or commercial impact. We used a Spartan 3AN XC3S700AN FPGA platform (Xilinx, USA) and utilised an external USB controller (FT2232H Mini-Module, FTDI, UK) to transfer the resulting fluorescence lifetime data to a standard PC. The FPGA and USB controller can be purchased for a total of ~£200. This specific FPGA platform offers flexibility in the design (e.g. the excitation frequency can either be generated on board or externally). It also imposes some constraints, of which the most relevant is the frequency range that can be generated using the on-board frequency synthesizers that spans 5 to 320 MHz and is limited by the bandwidth of the components. Although this range covers the excitation frequencies typically used in fluorescence lifetime measurements (i.e. from 5 to 80 MHz), it limits the number of temporal detection windows - particularly impacting measurements at higher excitation frequencies - and therefore the temporal resolution of the system.

The lower the value of *f*_*cc*_, the smaller is the phase shift between *f*_*s*_ and *f*_*exc*_ for each excitation period, and therefore the higher can be the temporal sampling density. For convenience and ease of implementation in the FPGA, the value of *f*_*cc*_ should be an integer fraction of *f*_*s*_, i.e.

2 $$ {f}_{cc}= k\ {f}_s $$

where *k* is an integer. *k* defines the number of sampling periods that fulfil a single cross-correlation period, thus also defining the number of bins in the fluorescence lifetime histogram. From Equations 1 and 2, we can derive the relation between excitation and sampling frequencies as.

3 $$ {f}_s=\frac{k}{k-1}\;{f}_{exc} $$

To implement multiple detection windows, we generate a clock running at a frequency *f*_*w*_ that is phase-locked at a multiple of *f*_*s*_, as described by Equation 4, where *n*_*w*_ defines the number of detection windows that fulfil a single sampling period, i.e.

4 $$ {f}_w={n}_w{f}_s $$

Equations 3 and 4 can then be used to write *f*_*w*_ as function of the excitation frequency *f*_*exc*_ *.*

5 $$ {f}_w=\frac{n_w k}{k-1}\;{f}_{exc} $$

This relationship, which can be implemented in the FPGA using the on-board frequency synthesizers (e.g. *f*_*s*_ can be generated from frequency division of *f*_*w*_), limits the number of detection windows that can be generated, due to constraints limiting *f*_*w*_ in the FPGA. For example, for an 80 MHz laser repetition rate and 256 bins (*k* = 256) in the fluorescence lifetime histogram, a frequency *f*_*w*_ equal to 321 MHz would have to be generated to realise 4-window detection architecture (*n*_*w*_ = 4), which is beyond the maximum operating frequency of the FPGA used in this implementation, i.e. 320 MHz. For our measurements, we used excitation at 20 or 40 MHz, with *n*_*w*_ = 4 and *k* = 256 (or *n*_*w*_ = 8 and *k* = 64, at 20 MHz excitation, only) architectures.

To build the fluorescence lifetime histogram, we keep track of the temporal detection window, *w*_a_, at which each single photon is read out and the corresponding phase offset, *φ*, between sampling and excitation frequencies at the time of the detection. Since *f*_*w*_ evenly divides a sampling period into *n*_*w*_ windows, it can be used as input for a counter that identifies each window of arrival within that period with a number ranging from 0 to *n*_*w*_ – 1. To keep track of the phase shift between *f*_*exc*_ and *f*_*s*_, an additional counter is implemented at the timescale of the sampling period. These values are then used to calculate the bin of arrival *t*_*ph*_ for each photon, which in our case is defined as shown in Equation 6. Finally, the value of *t*_*ph*_ for each photon is immediately transferred to the USB chip and then to the PC for further processing.

6 $$ {t}_{ph}\left({w}_a,\varphi \right)=\left[\left( k-1\right)-\varphi +\frac{w_a k}{n_w}\right] \mod (k) $$

## Home-Made Constant Fraction Discriminator

**Fig. 2:**
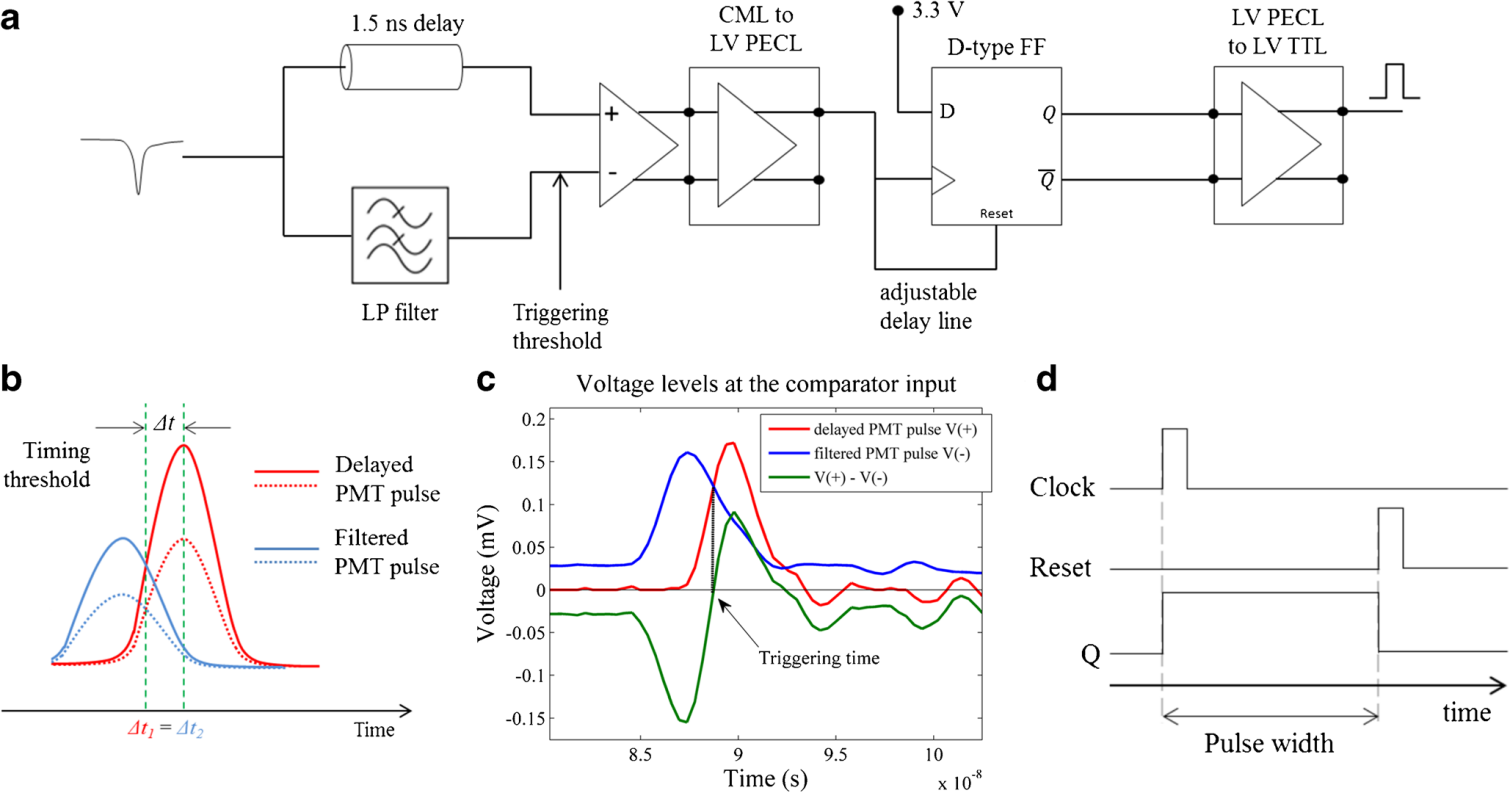
**a** Simplified diagram of our homemade CFD implementation. **b** CFD methodology illustrated for two pulses of different amplitude (solid and dashed red lines). Blue lines represent filtered version of the original PMT pulses. **c** Voltage levels measured at the comparator input (blue and red curves). Triggering occurs when the when the voltage of the two signals is the same. **d** Timing diagram for pulse stretching. The pulse width was set to 35 ns. The green dashed vertical lines indicate the time difference between the trigger of the comparator and the peak amplitude of the unfiltered pulse

In time-critical photon-counting applications, such as fluorescence lifetime measurements, it is essential to have a standardised approach to detect the arrival time of each photon. This can be realised using constant fraction discriminators (CFDs) that indicate the centre of the electronic pulses reporting each photon. While CFD circuits are relatively straightforward to implement at low (~MHz) frequencies using low-cost electronic components and well-established circuitry, operation at the high frequencies required for detectors with sub-nanosecond rise and fall times is critically dependent on the circuit design. This can be impacted by factors such as transmission line propagation and crosstalk, impedance mismatching, connector properties, delay, attenuation and ground inductance, amongst others [36], and such high-speed electronic circuitry is relatively complex and expensive. Commercially available CFDs typically cost ~£2000 for a single channel unit - and therefore are not suitable for a low-cost instrument, particularly if multichannel detection is required. To overcome this limitation, we have developed an analogue CFD that uses a simplified design to reduce the number of electronic components used. Discrimination at a constant fraction of the pulse amplitude is achieved by comparing a PMT pulse with a filtered version of the same pulse. Because the two signals are proportional, variations in pulse amplitude will affect both equally (see Fig. 2a).

In our implementation (see Fig. 2a), the PMT pulse is split in two lines: one is delayed and fed into the non-inverting input of a comparator (V_+_) and the other is low-pass filtered and fed to the inverting input (V_−_). The low-pass filter will attenuate and temporally-broaden the signal and introduce a delay. The delay of the first line is then adjusted (1.5 ns delay in our circuit) so that the pulses overlap and that the amplitude of the point of inflexion of the rising edge of the unfiltered pulse at the comparator is equal to the amplitude of the filtered pulse close to its peak, as illustrated in Fig. 2b. The temporal broadening of the filtered pulse allows the length of the delay line to be set relatively coarsely compared to a conventional CFD design without filtering. With this configuration, the comparator will effectively “see” a signal that is initially negative and that then becomes positive following a fast transition (see Fig. 2a, *green curve*). The comparator will trigger as the delayed pulse becomes greater than the filtered pulse, i.e. when V_+_ > V_−_. To avoid random triggering due to noise, a small positive voltage offset can be added to the inverting line. In contrast to a conventional CFD implementation, the PMT pulse is not fed onto a leading-edge discriminator for comparison against a voltage threshold. In our circuit, such discrimination is directly implemented by adjusting the voltage offset of the low-pass filtered signal. This approach reduces the number of electronic components used and the complexity of the circuit, as there is no need to match the delay of the comparator that produces triggering at a constant fraction of the amplitude with a leading-edge output line. Upon triggering, the comparator outputs a differential current-mode logic (CML) pulse of width equivalent to the time that the delayed pulse is greater than the attenuated pulse, which is approximately 1 ns for a cooled PMC-100-1 (Becker and Hickl GmbH, Germany).

In terms of FPGA triggering circuitry, there are two problems with this circuit: 1) the FPGA input ports do not support CML logic; 2) the 1 ns pulse width is not sufficient to trigger an event in the FPGA. To address the first issue, a voltage level translator was added to the circuit, to convert the signal from CML to low-voltage positive emitter-coupled logic (LV PECL). Pulse stretching was achieved using a D-type flip-flop: the positive LVPECL was fed to both the clock and reset input lines of the flip-flop and the D-input was constantly fed with a DC voltage of 3.3 V. Upon arrival of the signal at the clock input, the output of the flip-flop is set to a high state and will stay there until the delayed signal in the reset line brings the output to a low state. By adjusting the delay between clock triggering and reset, one can control the output pulse width. The timing diagram for this pulse stretching method is presented in Fig. 2d. Finally, the differential output of the flip-flop is converted to a single-ended signal for convenience, in this case low-voltage TTL (LV TTL).

## Characterization of the Detection Methodology

In order to validate the time-resolved detection system presented here, it was tested with a well-characterised cuvette-based time-resolved spectrofluorometer previously developed in our laboratory [35], where it could replace the “conventional” TCSPC detection system. A diagram of this setup is provided in supplementary material. In brief, this instrument comprises a gain-switched laser diode (LDH-P-C-375B, PicoQuant GmbH, Germany) providing 70 ps pulses at 375 nm with a maximum output power of 3.3 mW and adjustable repetition rate from 5 to 80 MHz, which was fixed to 40 MHz for the experiments described below. Excitation light from the laser was directed to a computer controlled shutter (SH05 shutter & SC10 controller, Thorlabs, Germany) and a rotatable filter wheel (Newport, USA) containing a set of neutral density filters with variable optical transmissions to allow adjustment of the beam intensity. Finally, the light was passed through a Glan-Taylor polarising cube and directed onto a quartz cuvette containing the sample. Fluorescence or scattered light from the sample were collected at right angles relative to the excitation beam, passing through a rotatable linear sheet polariser (all measurements were realised at the magic angle relative to the excitation light polarisation to suppress contributions from fluorescence anisotropy) and a motorised monochromator (CM110, CVI Inc., USA) for emission wavelength selection before being directed to a cooled photon-counting PMT (PMC-100-1, Becker and Hickl GmbH, Germany). For time-resolved measurements with our FPGA-based approach, the output signal from the PMT was directed to the homemade CFD and then routed to the FPGA. For TCSPC measurements, the PMT was directly connected to a TCSPC acquisition card (SPC-730, Becker and Hickl GmbH, Germany). All fluorescence lifetime data were analysed using *FLIMFit* [8], an open source package for analysis fluorescence lifetime data developed by our laboratory.

## Characterization of the CFD

### Timing Uncertainty

The timing uncertainty in a CFD predominantly results from fluctuations in the input pulse amplitude. Although, in principle, triggering occurs at a constant fraction of the maximum amplitude for each pulse, in practice this requires the pulse shape to be approximately constant from pulse to pulse. In particular, fluctuations in pulse rise times and FWHM will present corresponding variations in pulse bandwidth that would result in different attenuation and delay parameters in the low-pass filter. This would lead to fluctuations in the triggering time, *t*_trigger_, which can be defined as the time difference between the maximum amplitude of the delayed signal and the time point where the delayed and attenuated signals have identical voltages (Δ*t* in Fig. 2b). A further contribution to timing jitter could arise if the parameters of the low-pass filter that define the pulse attenuation are set such that the amplitude of the attenuated signal is so low that it is comparable to the amplitude of the noise. Fast photon-counting PMTs used in fluorescence lifetime measurements have rise-times typically below 1 ns [37], which is equivalent to bandwidths greater than 350 MHz. We found that a low-pass filter with cut-off frequency of 723 MHz resulted in an uncertainty of less than 5% in the triggering time of PMT pulses (Δ*t* in Fig. 2b) with less than 1 ns rise-time and we used this in our CFD implementation.

### Voltage Offset

**Fig. 3:**
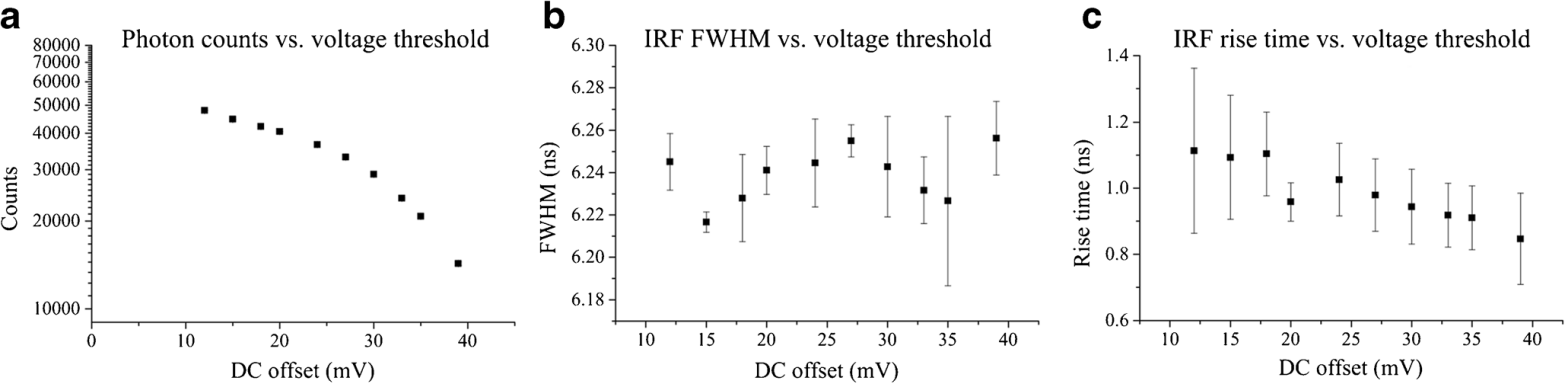
System dependence on the discriminator offset voltage: **a** photon counts; **b** IRF FWHM; **c** IRF rise time. Measurements were realised using 375 nm excitation light at 40 MHz, with 4 detection windows and 256 bins

True triggering at a constant fraction of the pulse amplitude is achieved when the DC component at each comparator input is zero. In practice, however, this will cause the comparator to be triggered by noise or artefacts, such as reflections in the circuit, as well as by photodetection events. A slightly positive voltage offset is therefore needed at the inverting input (V_−_) such that the voltage at this point is always higher than in the non-inverting input except when a true photodetection signal arrives. The voltage offset slightly above noise yields the best results. As the offset voltage is increased, the triggering time will start to depend on the pulse amplitude and the number of detected photon counts will decrease. Thus the offset voltage should ideally be set just above the noise level. To investigate the influence of the offset voltage in our CFD, we measured the fluorometer instrument response function (IRF) using Ludox beads (420786-1 L, Sigma-Aldrich, Canada), at different voltage offsets for pulsed excitation at 40 MHz, with a 4-window detection architecture and 256 bins, which yields a gate width of ~6.25 ns. The IRF FWHM and rise time are presented in Fig. 3 as a function of offset voltage with the corresponding photon counts.

For offset voltage thresholds below 10 mV, triggering due to noise becomes predominant and the IRF is not measurable. At higher offset voltages, the detected photon count decreases as fewer pulses reach the minimum voltage that triggers the comparator (Fig. 3a) and the IRF approximates to the shape of the detection gate of 6.25 ns (Fig. 3b). The rise time of the IRF (~1 ns, Fig. 3c) is set by a combination of timing uncertainty in the clock generation and inherent jitter in the FPGA logic together with the CFD jitter, which is degraded by pre-pulsing effects in the PMT. Pre-pulsing occurs for a small fraction of the incident photons and can generate a low amplitude pulse that appears before the real photodetection event if the threshold is too low. In TCSPC, pre-pulses can be avoided by setting the detection threshold above the pre-pulsing amplitude. Our method is more sensitive to pre-pulsing artefacts as the detection threshold is implemented directly at the comparator input and therefore its value is adjusted to minimise pre-pulsing effects while maximising the real photon count rate. Altogether, due to the long detection windows in our implementation, pre-pulses and real photodetection events are undistinguishable, which translates in longer rise times at lower voltage offsets, as demonstrated in Fig. 3c. Following these experiments, we selected an offset voltage of 18 mV and this value was used in all subsequent experiments.

## Fluorescence Measurements of Reference Fluorophores

**Stilbene-3** is a monoexponential dye with a reported fluorescence lifetime of 1.2 ns in water [38] and therefore represents a convenient test sample for our system. A solution of 50 μM Stilbene-3 in purified water at room temperature was measured with excitation at 375 nm. The detection channel was centred on 460 nm, which is close to the measured fluorescence emission peak of this dye (data not shown). The fluorescence lifetime determined from our conventional TCSPC acquisition was 1.21 ± 0.01 ns (*n* = 3), which is consistent with the lifetime values reported in literature. We investigated the performance of the FPGA-based time-resolved detection system at different excitation intensities using the fluorometer described above.

**Fig. 4:**
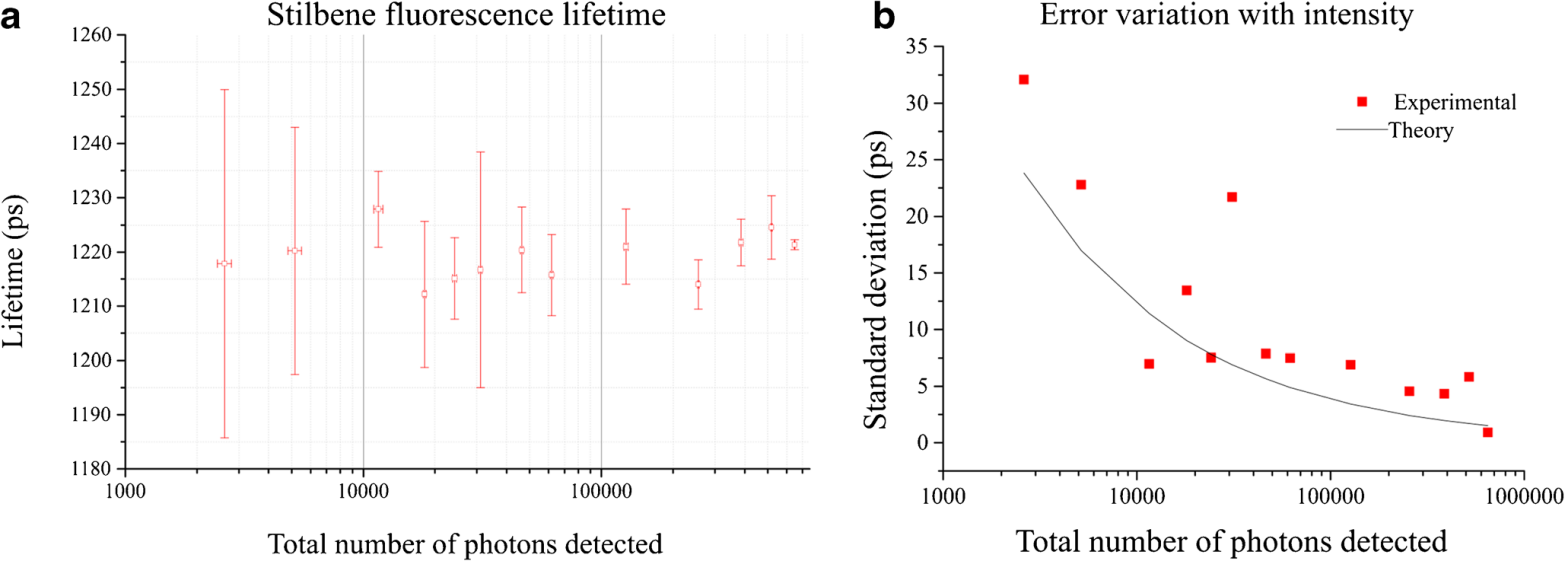
**a** Fluorescence lifetime of Stilbene measured at different excitation intensities (*n* = 3 for each data point); **b** Error variation with total intensity compared to the theoretical curve for an ideal TCSPC system. Measurements were realised using 375 nm excitation light at 40 MHz using the low-cost system with a 4-window architecture and 256 bins in the fluorescence histogram. Error bars in (*a*) show the standard deviation

In Fig. 4a, we observe that the fluorescence lifetime measured for Stilbene-3 is independent of the total number of photons collected. If we average all measurements, we obtain a lifetime of 1.22 ± 0.01 ns, which is in agreement with the lifetime value measured with conventional TCSPC. The precision of our instrument as a function of the number of photons detected is presented in Figure 4b, where we compare the experimental standard deviation (red squares) with the standard deviation (black line) calculated from theory for an ideal TCSPC system: $\sigma =\tau /\sqrt{N}$ [26, 39, 40], where *N* denotes the total number of photons detected in each acquisition. The plot in Fig. 4b shows that the uncertainty in the fluorescence lifetime measurement follows the expected trend with the total number of photons detected.

**Fig. 5:**
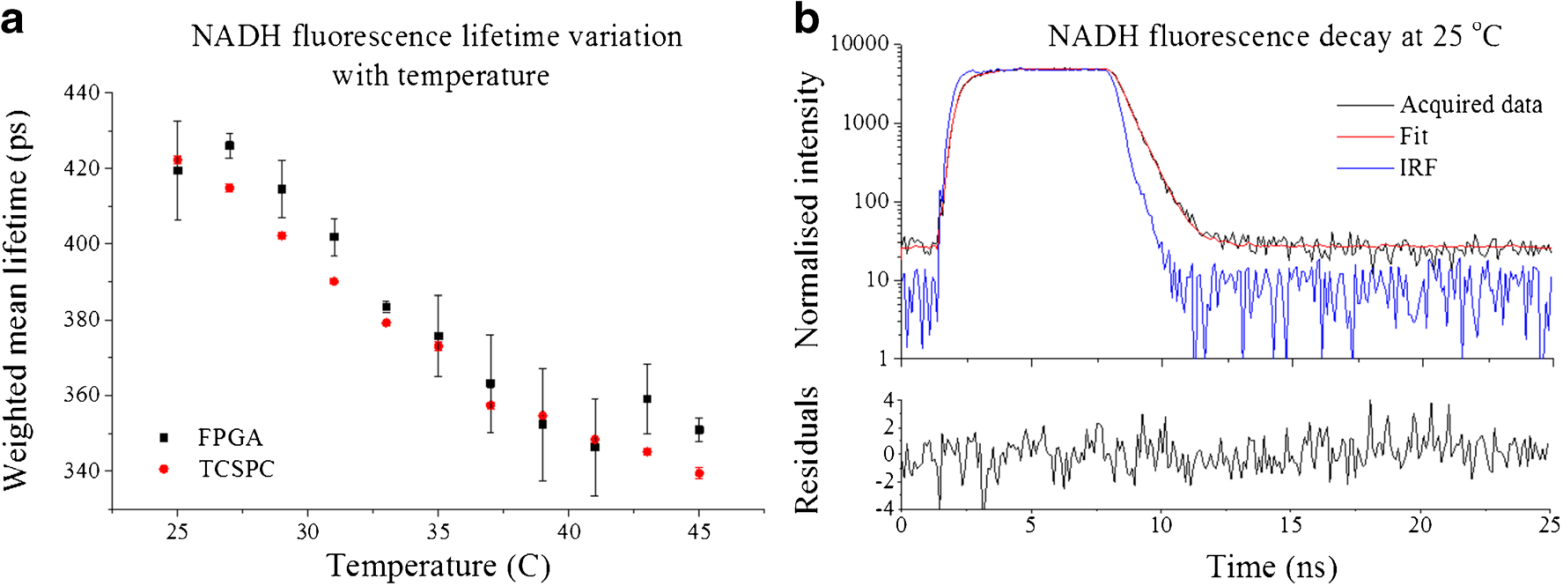
**a** Variation of NADH mean fluorescence lifetime with temperature for TCSPC and FPGA detection methods; **b** fluorescence decay of NADH at 25 °C, measured with the FPGA detection methodology. Measurements were realised at 460 nm using 375 nm excitation light at 40 MHz with a 4-window architecture and 256 bins in the histogram. Approximately 500,000 photons were collected for each measurement. Error bars in (A) show the standard deviation

**NADH** is an important test sample because of its contribution to cellular autofluorescence and its potential to report on cell metabolism**.** It presents a complex fluorescence decay profile with a relatively short mean fluorescence lifetime (450 ps [41]) that can change with temperature [42]. NADH therefore provides a suitably challenging test sample to evaluate our instrument and we can exploit the temperature dependence to probe its ability to resolve small lifetime changes. Figure 5a presents fluorescence lifetime measurements of NADH made at different temperatures using both conventional TCSPC and the low cost FPGA system. The intensity weighted mean fluorescence lifetime measured with FPGA-based detection is in reasonable agreement with the conventional TCSPC measurement and, although the precision of the FPGA measurements is lower than for the conventional TCSPC system, these results show that the FPGA-based system can resolve fluorescence lifetime variations of 10s of picoseconds - even with much larger (6.25 ns) detection windows.

## A Complete low-Cost Time-Resolved Fluorometer

**Fig. 6:**
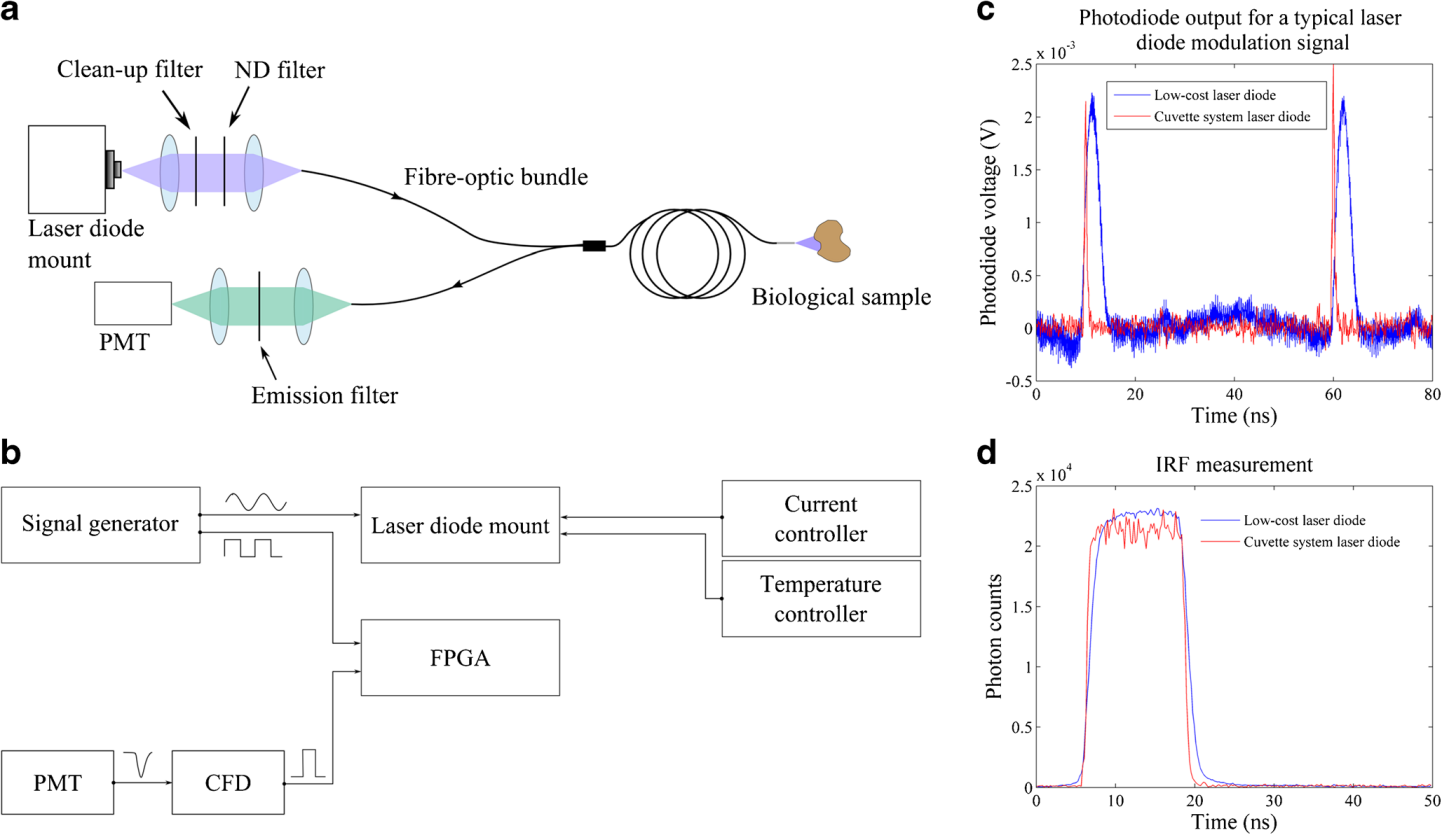
**a** Optical layout and **b** electronic configuration of the low-cost single-point time-resolved fluorometer. **c** Typical optical output of our modulated laser diode (in blue, FWHM =3.07 ± 0.36 ns) and output from the commercially available laser diode (in red, FWHM =0.48 ± 0.06 ns) employed in the cuvette-based system [24]. For both measurements, light was delivered to the photodiode using a fibre-optic probe. **d** Measured IRF profiles of the FPGA-based detection system obtained with scattering Ludox beads, yielding FWHM of 12.50 ± 0.09 ns for the low-cost system and 12.35 ± 0.04 ns for the cuvette-based system with commercial picosecond diode laser

The previous section indicates that the low-cost detection approach compares reasonably well with the “gold-standard” TCSPC detection system using the same picosecond pulsed excitation laser and cooled photon-counting PMT (which provides a low dark count rate and low after-pulsing). However, these components have a combined cost of approximately £15,000 and therefore still present a significant cost that could hinder some potential applications. We therefore explored the implementation of a complete low-cost time-resolved fluorometer incorporating a less sophisticated pulsed laser diode source for excitation and an uncooled photon counting PMT together with the FPGA-based detection system. The optical layout of this instrument, which includes a fibre-optic probe for in situ measurements, is presented in Fig. 6a. Excitation light was provided by a single-mode laser diode (Changchun New Industries, China) with centre wavelength at 405 nm and maximum output power of 100 mW in CW operation, which was fitted on a temperature-controlled laser diode mount (TCLDM9, Thorlabs, USA) providing suitable interfaces for external RF modulation and current control. To achieve pulsed excitation, the laser diode voltage was modulated with a 20 MHz sinusoidal waveform (Vpp ≈ 8 V, 50 Ω output) provided by a benchtop function generator (TG2001, Thurlby Thandar Instruments Ltd., UK) combined with a DC voltage via a bias-tee network that exists in the diode mount. Short optical pulses were achieved by fine-tuning the DC current so that the laser diode current was only above threshold for a small fraction of the modulation period. The optical power at the sample was adjusted using neutral density filters in the excitation path and the beam was directed into a fibre-optic probe, identical to that described in a previous study [21], featuring three optical fibres used to deliver excitation light and fourteen optical fibres used to collect the fluorescence signal. A lens relay directed the emission light used to the photocathode of an uncooled photon counting PMT (PMH-100, Becker and Hickl, Germany) via a band-pass filter (FF03–525/50–25, Semrock, USA) to remove any residual excitation light and to restrict fluorescence detection to the range of wavelengths between 500 to 550 nm. Finally, the output signal of the PMT was converted into a logic pulse using our homemade CFD and then fed to the FPGA input port to realise time-resolved detection. To maintain synchronicity, the FPGA was fed with a TTL-based clock at 20 MHz that was used as a reference clock to generate the sampling windows and was derived from the same function generator as the laser diode modulation signal. A diagram of the electronic configuration of this system is shown in Fig. 6b.

Figure 6c shows a typical optical pulse from the 20 MHz modulated diode laser, measured using a fast photodiode (SV2-FC, Thorlabs, USA) after propagating through 1.3 m of multimode optical fibre-optic bundle (NA = 0.22). These measurements include the temporal broadening due to modal dispersion in the fibre, which was expected to be ~130 ps. The excitation pulses have a deconvolved FWHM of 3.07 ± 0.36 ns and rise time (10 to 90% of the pulse amplitude) of 1.52 ± 0.08 ns. The corresponding IRF of the low-cost system is shown in Fig. 6d (in blue), for which we calculated a FWHM of 12.50 ± 0.09 ns with 2.35 ± 0.21 ns rise time. For comparison, similar measurements were made using the 375 nm excitation laser diode installed in the cuvette-based fluorometer following propagation through its 2 m of multimode fibre-optic extension [35] that guides light to and from the sample. For this laser diode, we measured an excitation pulse FWHM of 0.48 ± 0.06 ns with rise time of 0.25 ± 0.05 ns using the fast photodiode. The corresponding IRF measured with the FPGA-based detection electronics and uncooled PMT was 12.35 ± 0.04 ns FWHM with 1.74 ± 0.08 ns rise time. These results show that even though the excitation pulses from our modulated laser diode are significantly longer than those provided by the commercial laser diode, this translates essentially in longer IRF rise and fall times but a similar IRF FWHM for the system (Fig. 6d).

## Fluorescence Lifetime Measurements of Reference Fluorophores

### Single Exponential Decays

The temporal accuracy of the low-cost instrument compared to the cuvette-based time-resolved spectrofluorometer by measuring a number of fluorescent dyes presenting single exponential decay characteristics. In both instruments, emission was detected at 525 nm excited by 405 nm pulses at 20 MHz. In the cuvette-based instrument this was provided by a supercontinuum laser (SPC-400, Fianium, UK) installed in the system. The IRF for each instrument was measured at the excitation wavelength using a scattering sample of Ludox beads. Measurements in the low-cost system were made using two different detection architectures, identified as “architecture 1” (4 detection windows and 256 bins) and “architecture 2” (8 detection windows and 64 bins). For the cuvette-based system, TCSPC data were acquired using 1024 time bins. Five measurements of the sample were made with each instrument, for which the results are presented in Table 1.

**Table 1:** Average lifetimes of reference fluorophores in solution measured using the cuvette-based and low-cost instruments. A total of 5 acquisitions were realised for each fluorophore. All measurements were realised at room temperature. All values are reported in nanoseconds (ns). The total number of photons detected was approximately 1,000,000 in the cuvette based system and 500,000 in the low-cost system

Fluorophore	Cuvette-based system	Low–cost system	
		Architecture 1	Architecture 2
Coumarin 6	2.61 ± 0.01	2.64 ± 0.04	2.59 ± 0.02
Coumarin 153	4.68 ± 0.01	4.72 ± 0.05	4.72 ± 0.04
Coumarin 307	5.32 ± 0.01	5.38 ± 0.02	5.37 ± 0.03
Coumarin 314	3.52 ± 0.01	3.58 ± 0.05	3.53 ± 0.04
Fluorescein	4.06 ± 0.01	3.91 ± 0.03	4.10 ± 0.07

Overall, the fluorescence lifetimes extracted for these fluorophores are in reasonable agreement between instruments, including between the two different detection architectures of the low-cost system. These results demonstrate that it is possible to resolve single exponential decays with temporal accuracy comparable to TCSPC yet using nanosecond-wide excitation pulses. It should be noted that potential artefacts arising from fluorescence anistropy were precluded for the cuvette-based system since these measurements were made with vertically polarised excitation light and a detection polarizer at the magic angle. This was not the case for the measurements with the low-cost system.

### Double Exponential Decays

**Fig. 7:**
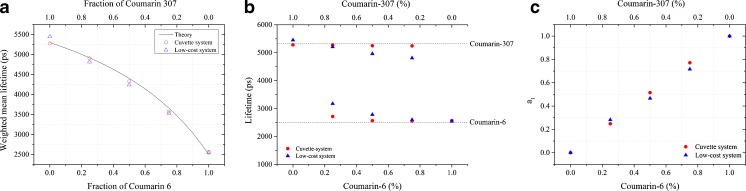
**a** Intensity weighted mean fluorescence lifetimes of Coumarin 6 and Coumarin 307 solutions and comparison with values expected from theory. Data of pure solutions were fitted to a single exponential decay model, while data of mixture solutions were fitted to double exponential decay models. The total number of photons detected was approximately 2,000,000 in the cuvette based system and 350,000 in the low-cost system. **b** Fluorescence lifetimes extracted from Coumarin 6 and Coumarin 307 measured with both instruments. Dashed lines represent the expected fluorescence lifetimes the pure compounds in ethanol (τ_C6_ = 2.5 ns; τ_C307_ = 5.3 ns). **c** Relative proportion calculated for Coumarin 6 (a_1_/(a_1_ + a_2_)) from the fitting results

To investigate the ability of our instrument to resolve double exponential decay profiles, solutions containing mixtures of Coumarin 6 (C6, *τ* = 2.5 ns) and Coumarin 307 (C307, *τ* = 5.3 ns) with different relative proportions were measured using both instruments, together with measurements of the pure compounds. For mixtures of the dyes, their relative proportion was adjusted by fixing C6 concentration at 6 μM and varying the concentration of C307 in each solution. Concentrations of the stock solutions of both dyes were calculated from absorbance measurements in a spectrophotometer (UV-3101PC, Shimadzu, Japan) using molar extinction coefficients of 54 × 10^3^ cm^−1^/M at 460 nm for C6 and 20 × 10^3^ cm^−1^/M at 460 nm for C307 [43]. In the low-cost system, time-resolved detection was implemented using 4 detection windows and 256 bins, which corresponds to a temporal bin of ~195 ps. In the cuvette-system, TCSPC was implemented using 4096 bins, which corresponds to a bin width of approximately 12 ps at 20 MHz. Figure 7a shows the fluorescence intensity decay profiles of the dye solutions measured in the low-cost system and Fig. 7b presents the intensity weighted mean lifetimes obtained from measurements with both instruments, including a comparison with the expected values. The mean fluorescence lifetimes measured with the low-cost instrument agree closely with those measured in the cuvette-system using TCSPC detection. With respect to the relative contributions of C6 and C307 to the fluorescence decays, the results show that the low-cost system can resolve the lifetime and fractional populations of both fluorophores, albeit with lower accuracy than TCSPC measurements. The individual fluorescence lifetimes of C6 and C307 are overestimated and underestimated, respectively, when the contributions of these fluorophores to the fluorescence decay is low (Fig. 7b). This error is less apparent in TCSPC data. The number of photons detected during the acquisition of the data in the low-cost instrument (approximately 3.5 × 10^5^ photons, on average) is lower than with TCSPC acquisition (approximately 2.0 × 10^6^ photons, on average), which results in a lower SNR that may reduce the sensitivity of the system to resolve complex decays and may also explain differences between the two instruments.

## Application to Tissue Autofluorescence

**Fig. 8:**
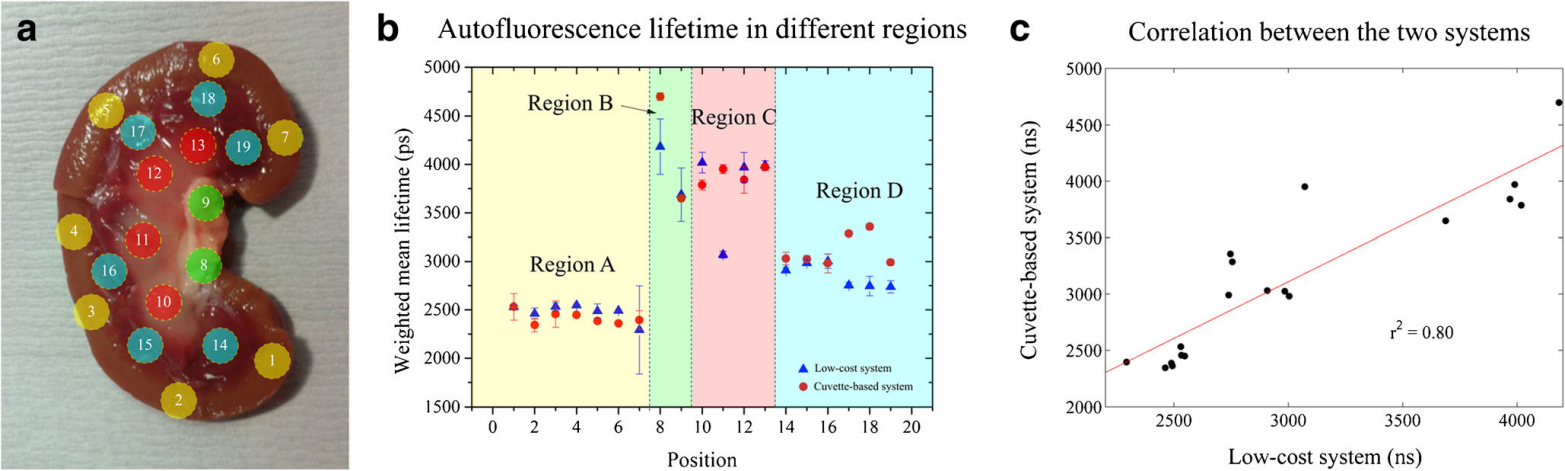
**a** Photograph showing the spatial locations of the measurements of the internal structure of a lamb kidney. Each region is identified by a number and the colours show the region each measurement was taken in Fig. 8b. **b** Autofluorescence lifetime in different regions. Data show weighted mean fluorescence lifetime extracted from a double exponential fitting. **c** Correlation between the weighted mean fluorescence lifetime of the cuvette system and low-cost system for each position. A linear fit to the data output an r^2^ of 0.80 or, similarly, a Pearson coefficient r of 0.89

To investigate the ability of the low-cost time-resolved fluorometer to provide contrast between the complex autofluorescence signals excited in biological tissue, the autofluorescence profiles of fresh lamb kidney were measured using both the low-cost instrument and the cuvette-based TCPSC system. To this end a lamb kidney was bisected, revealing four different structures that can be easily identified macroscopically and which provide autofluorescence lifetime contrast due to their different biochemical and structural compositions [44–46]. Single point measurements were made via the fibre-optic probe at a number of locations indicated in Fig. 8a. A total of three measurements were taken in each location and for each single acquisition the probe was taken away from the sample and repositioned, such that the standard deviation of the measurements includes the impact of small changes in location.

The results presented in Fig. 8b show that both instruments reported different intensity-weighted mean lifetimes in different regions and the observed variations of lifetime were mostly similar for the two instruments. Where larger discrepancies are observed (e.g. at position 11), this may have resulted from the fibre-optic probe collecting autofluorescence from different locations in the tissue for the two systems, as the probe was repositioned between measurements. Figure 7c shows the correlation between the intensity-weighted mean autofluorescence lifetimes extracted at each position using each instrument. The Pearson coefficient *r* of 0.89 indicates a strong correlation between measurements at each position in the two systems.

## Conclusions

The wide-spread exploitation of autofluorescence lifetime measurements for clinical and other applications requires more compact and lower cost instrumentation compared to the current state-of-the-art. Here we presented a low-cost time-resolved fluorometer integrated with a fibre-optic probe that can be applied to resolve lifetime changes for the vast majority of commonly used fluorescent labels and for the most commonly studied endogenous fluorophores in biological issue. This capability was illustrated with exemplar fluorophores and unlabelled biological tissue and was validated by a comparison against a well-characterized “gold-standard” spectrofluorometer based on a commercial picosecond excitation laser and TCSPC detection system.

Our implementation utilizing a modulated laser diode for excitation and a simplified CFD together with FPGA-based circuity for time-resolved detection of the fluorescence signal could be constructed with a component cost of less than £1000. While this figure does not include the fibre-optic probe, it indicates that a time-resolved fluorometer suitable for clinical studies could be constructed with a total component cost of less than £5000. This figure could be significantly reduced through mass production.

Overall, these results demonstrate that our low-cost instrument can read out intrinsic contrast in biological tissue by means of autofluorescence lifetime, which is demonstrated by the small errors in the measurements for this system, although the precision is lower than that achieved using the cuvette-based TCSPC system.

Clearly the instrument could be further optimised and extended, e.g. to provide multispectral time resolved detection or incorporate other modalities such as optical coherence tomography or spectrally resolved reflectometry (e.g. for oxygenation measurements or elastic light scattering). The time resolution could be improved by replacing the FPGA with one providing a higher maximum operating frequency than 320 MHz, although this would make the design and programming more challenging and could increase the cost. In its present configuration, we believe that this low-cost time-resolved fluorometer combined with a fibre-optic probe has significant potential for clinical studies and could aid diagnosis of diseases including cancer, heart disease and osteoarthritis [12–19, 22], including for intraoperative provide guidance during surgical procedures.

## Electronic supplementary material

ESM 1 (DOCX 126 kb)
