# Supplementary material for: Development of Low-Cost Instrumentation for Single Point Autofluorescence Lifetime Measurements
Source: J Fluoresc. 2017 May 25;27(5):1643–54. doi: 10.1007/s10895-017-2101-7 (PMC5583312; doi:10.1007/s10895-017-2101-7)
Supplement: Supplementary file 1 — (DOCX 126 kb) [file 10895_2017_2101_MOESM1_ESM.docx]

# Supplementary material


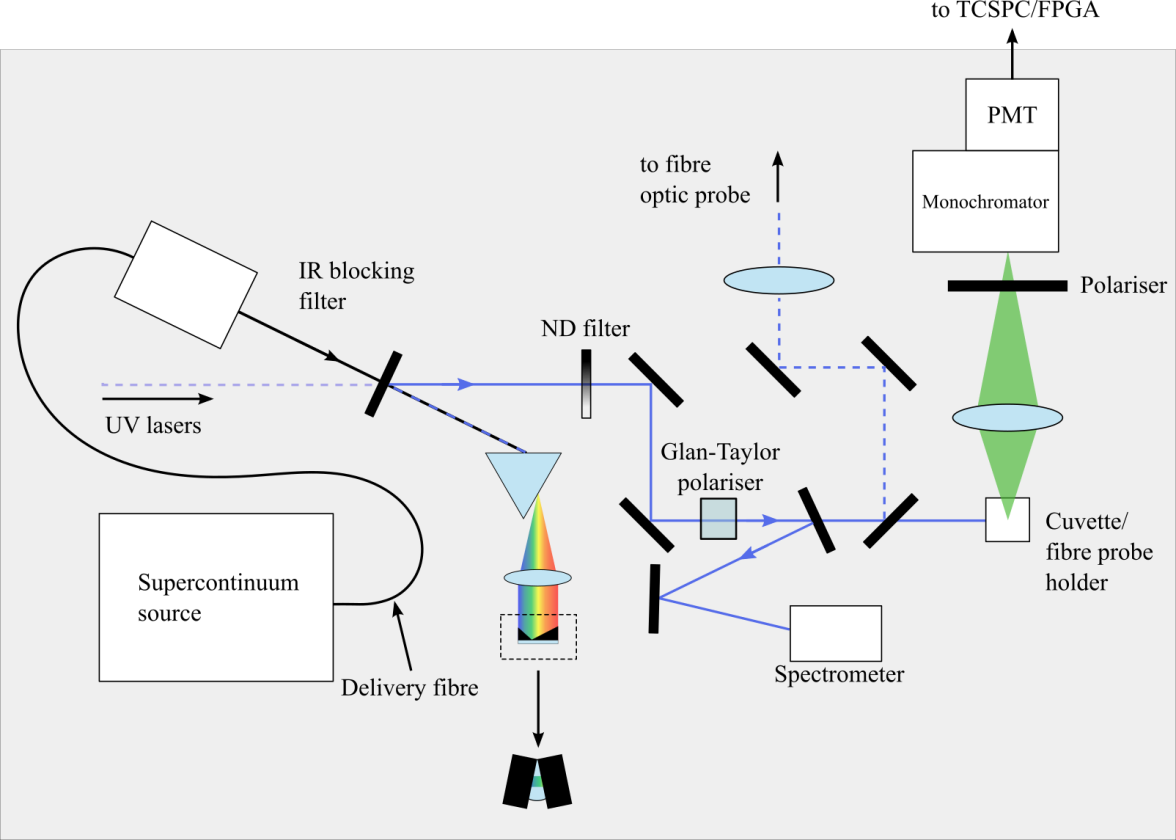


*Figure 1A. Optical layout of the cuvette-based time-resolved spectrofluorometer used to compare our low-cost instrument against a TCSPC system*
